# Hospital Frailty Risk Score Predicts Long-Term Hospital Readmissions in Very Old Patients with Heart Failure

**DOI:** 10.3390/jcm15072703

**Published:** 2026-04-02

**Authors:** Anna Giani, Francesco Fantin, Arianna Bortolani, Elena Zoico, Silvia Urbani, Alessandro Gavras, Giulia Guastalla, Rocco Micciolo, Mauro Zamboni

**Affiliations:** 1 Department of Medicine, Section of Geriatrics, University of Verona, 37100 Verona, Italy; 2 Centre for Medical Sciences—CISMed, Department of Psychology and Cognitive Science—DIPSCO, Section of Geriatric Medicine, University of Trento, 38068 Rovereto, Italy; 3 Centre for Medical Sciences, Department of Psychology and Cognitive Sciences, University of Trento, 38123 Trento, Italy

**Keywords:** frailty, hospital frailty risk score, heart failure, hospital readmissions

## Abstract

**Background**: Heart failure (HF) is a highly prevalent condition among older adults, with a remarkable rate of re-hospitalization due to exacerbations. Frailty is strongly correlated with negative outcomes and might reveal patients in need of tailored follow-up. The aim of the study is to evaluate the predictive role of frailty in long-term re-hospitalization risk. **Methods**: In this prospective study, older adults hospitalized in an acute geriatric ward due to HF were included. Frailty was assessed at admission time by hospital frailty risk score (HFRS). Any hospital readmission was investigated up to one year after discharge. Patients were divided into tertiles upon receiving their HFRS, and readmission risk was evaluated by Cox regression models. **Results**: Among 213 patients (mean age 86 ± 7 years, 98, 46% male), 155 were frail according to HFRS. A total of 117 subjects were readmitted to hospital within one year after discharge. A Cox regression model showed that, even after adjustment for multiple selected variables, there was a significant increasing trend in the readmission risk across HFRS tertiles: compared with the lowest tertile, patients in the middle tertile had an risk of readmission of 1.29 (95% C.I.: 1.03–1.61), while those in the highest tertile showed a further 1.29 increase. **Conclusions**: Among patients with HF, long-term re-hospitalizations might be largely influenced by a previous state of frailty. HFRS is a feasible and broadly applicable tool that could be included in routine clinical practice to promptly identify heart failure patients at higher risk of readmission, in order to plan tailored assistance measures.

## 1. Introduction

Heart failure (HF) is a frequent and remarkable cause of hospital admission and readmission among older adults, and in this setting, frailty appears to be an underestimated yet potentially detrimental prognosis modifier [1].

Each episode of acute HF is followed by a “vulnerable phase” of approximately 90 days, characterized by the greatest risk of mortality and re-hospitalization, mainly driven by recurrence of volume overload and congestion [2]. However, after the vulnerable phase, a period of relative clinical stability is usually described, in which any readmissions may have other triggering factors. Previous studies endorsed tailored HF therapeutic interventions to reduce hospital readmissions [3,4,5], but even with adequate adherence to guideline-based HF specific management strategies, the re-hospitalization rate remains considerable [6].

From this perspective, there is a compelling need for early identification of patients at higher risk of re-hospitalization both in the short and long term, to adopt tailored strategies after discharge.

Among several well-known predictors of hospital readmission [7] in HF patients, frailty plays a pivotal role, but different models and tools are currently applicable to estimate frailty status, providing different results in studies, with a relative lack of standardized tools to assess frailty in this particular population [8,9]; among hospitalized patients with acute decompensated HF, frail subjects are frequently misrecognized and not given appropriate post-discharge management [10]. Although the phenotypic model and the cumulative model apparently equally predict mortality, they are not always easily applicable to the clinical in-hospital setting [11].

Hospital frailty risk score (HFRS) is a frailty index developed by Gilbert and colleagues that relies on an algorithm based on ICD-10 (International Statistical Classification of Diseases and Related Health Problems, Tenth Revision) codes, which are considered a priori as possible frailty markers [12].

The aim of the study was to evaluate the predictive role of frailty, assessed by HFRS, in long-term (up to 1 year) re-hospitalization in a cohort of older patients hospitalized due to HF.

## 2. Materials and Methods

**Figure 1:**
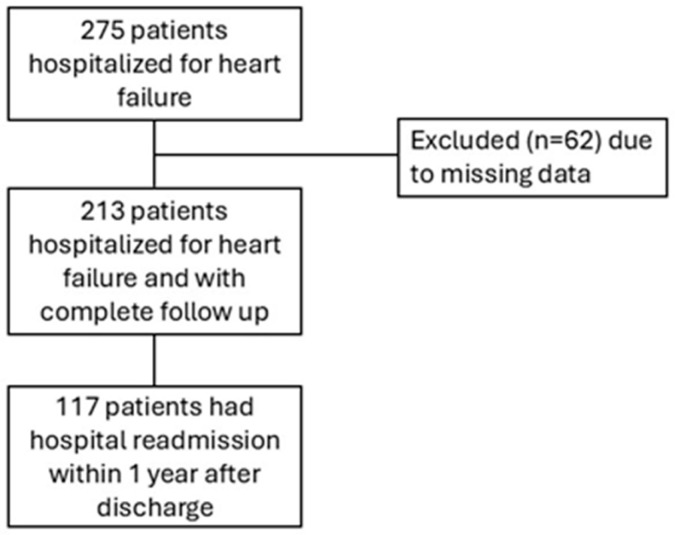
Flow chart of the study population.

In the present prospective study, all the patients aged over 65 years hospitalized in the Geriatric division of Verona University Hospital from September 2018 to December 2019 due to heart failure were consecutively enrolled. Exclusion criteria were readmission within 48 h from discharge and discharge to long-term care facilities. Among the 275 included patients, 62 were excluded due to missing data in electronic health records; thus, 213 subjects formed the study population (Figure 1).

These patients were prospectively enrolled upon admission and re-evaluated with a phone interview follow-up up to one year after discharge: the outcome was hospital readmission (within one year). All patients provided written informed consent; the study was approved by the local Ethics Committee.

Patients underwent a complete clinical evaluation, and medical history was recorded at admission.

Heart failure was diagnosed in the presence of clinical signs and symptoms (e.g., fluid overload, pulmonary crackles, peripheral edema, dyspnea, and fatigue), supported by suggestive thorax X-ray imaging and/or a natriuretic peptide increase. All the patients included were classified as NYHA classes 3 and 4. Echocardiography was performed by expert cardiologists, as required by clinical conditions. The ejection fraction was registered and deemed preserved if above 50%.

Frailty was estimated by HFRS, calculated as proposed by Gilbert and colleagues [12], evaluating pre-admission comorbidities (evaluated at the day prior admission) and assigning weights to 109 specific ICD-10 codes that are known to be associated with an increased risk of frailty. A specific weight was given to each included code, reflecting the extent to which the associated comorbidity contributes to frailty risk; the final score is obtained as the sum of the single weight scores. Frailty was defined by HFRS ≥ 5, as previously suggested [13,14]. Patients were classified according to tertiles of HFRS (first tertile HFRS ≤ 5, second tertile HFRS ≤ 5.63).

Upon admission, patients underwent a comprehensive geriatric assessment considering activities of daily living (ADL), instrumental activities of daily living (IADL), Barthel Index (BI), Geriatric Depression Scale (GDS); Mini Nutritional Assessment (MNA), mini mental state examination (MMSE), and Charlson Comorbidity Index (CCI).

At admission time, fasting venous blood samplings were obtained, and the following measurements were registered: blood count (Sysmex XN 9100, Kobe, Japan), NT-pro-BNP (Roche Cobas 8000, Monza, Italy), serum albumin (Roche Cobas 8000, Monza, Italy), and Creatinine (Roche Cobas 8000, Monza, Italy); the estimated glomerular filtration rate (eGFR) was calculated by the Cockroft–Gault formula.

Atrial fibrillation (AF) presence was investigated by 12-lead electrocardiography (MAC2000, GE Healthcare, Milwaukee, WI, USA) performed at admission time and considered present even in patients with anamnestic paroxysmal AF.

Variables are displayed as mean and standard deviation (SD) or proportions. Non-normally distributed variables were log-transformed prior to analyses. Male and female patients were compared using an unpaired *t*-test. A chi-square test was used when comparing categorical data between groups. The Cox regression model was employed to evaluate the effect of selected variables on the first readmission risk (which was considered the primary outcome); the independent variables were chosen based upon clinical relevance, and, namely, were HFRS categories (patients in the lowest tertile were considered the reference group), age, systolic blood pressure and NT-proBNP at admission time. Times of death were treated as censored observations. Proportional hazard assumptions were checked by the Schoenfeld test. To assess whether the effect of HFRS could be influenced by censoring due to death, a separate Cox regression model was fitted considering both the first hospital readmission or death (whichever occurred first) as outcomes and considering the same covariates.

A *p*-value ≤ 0.05 was considered statistically significant.

Analyses were performed using SPSS 23.0 version for Windows (IBM, Armonk, NY, USA) and R (https://www.r-project.org, version 4.4.3, 2025).

## 3. Results

A total of 213 patients, mean age 86 ± 7 years, of whom 98, 46% were males, were included the study population; the main characteristics are listed in Table 1.

Female patients were older, with major struggles in daily activities (ADL *p* < 0.001, IADL *p* = 0.02, BI *p* = 0.002) and in cognitive profile (MMSE, *p* < 0.001), along with major impairment in renal function (*p* = 0.022). There were 155 frail patients according to HFRS; mean HFRS was 6 ± 2, without significant difference between sexes. In the overall population, 117 patients were readmitted to hospital within one year after discharge (without difference between sexes). None of the study subjects were discharged to long-term care facilities, neither were any readmitted within the first 48 h after discharge. When considering patients with and without frailty (HFRS threshold of 5), a numerical yet not statistically significant difference was observed in terms of readmissions: 89 frail subjects, 57% of all frail individuals vs. 28 non-frail patients, and 48% of non-frail ones. Within the observation time (one year after discharge), 84 patients died, of whom 32 died without reporting any hospital readmission.

A Cox regression model fitted employing only HFRS category as an independent variable showed an increasing risk of readmission across HFRS categories: compared with patients in the reference (lowest tertile) group, the hazard rates of readmission were 1.34 and 1.67 in the second and in the third group, respectively. The trend was statistically significant (Likelihood Ratio Test = 5.55; *p* = 0.018). This result was confirmed also after having evaluated the effect of HFRS adjusted for age, SBP at admission, and nt-proBNP, which were considered as clinically important variables for readmission risk (Likelihood Ratio Test = 4.95; *p* = 0.026). Table 2 shows the results of the Cox regression model, which considered as covariates age, SBP at admission, nt-proBNP and HFRS. Even after adjustment, an increasing trend in readmission risk was observed across HFRS tertiles: compared with the lowest tertile, patients in the middle tertile had a risk of readmission of 1.29 (95% C.I.: 1.03–1.61), while those in the highest tertile showed a further 1.29 increase. The Schoenfeld test did not provide evidence of non-proportional hazards.

A separate Cox regression model, employing the same covariates in Table 2, was finally fitted considering both the first hospital readmission or death (whichever came first) as outcomes. The results were quite similar to the previous one. After adjustment, an increasing trend in readmission risk was confirmed across HFRS tertiles (Likelihood Ratio Test = 6.75; *p* = 0.009): compared with the lowest tertile, patients in the middle tertile had a risk of readmission of 1.30 (95% C.I.: 1.07–1.59), while those in the highest tertile showed a further 1.30 increase.

## 4. Discussion

The main finding of the present study is that frailty, measured by HFRS, is a significant predictor of long-term hospital readmission among patients with HF, widening the perspective on readmission predictors [7] and considering the complex network of interconnected conditions characterizing the older adult with HF.

Hospital readmissions are a major issue among older adults, and the role of frailty has attracted significant attention in recent years. Despite the clinical diagnoses that led patients to hospitalization, previous studies tested different frailty definitions to investigate the predictive role of frailty in short-term re-hospitalization [15].

In the particular setting of HF, it is acknowledged that despite appropriate adherence to guideline-directed management strategies, re-hospitalization rates remain substantial [6], and this generates a compelling need for the early identification of patients at increased risk of re-hospitalization, in order to implement individualized post-discharge management strategies.

Frailty is a cornerstone of the multidimensional evaluation of HF patients [16,17,18] and generally associated with negative outcomes [19,20,21,22,23]. Previous studies showed that both the frailty phenotype [21,23] and different cumulative models [20,22] are predictors of mortality, hospitalization burden, and poor prognosis in patients with HF; moreover, they outlined a positive association between baseline frailty and hospital readmissions [21,22]. However, despite significant consistency in the results, showing frailty associated with negative outcomes, there is a relative methodological heterogeneity among studies, and a unique and standardized measurement of frailty is still lacking.

Among different frailty tools, since based on a reproducible calculation on electronic health records, HFRS has attracted increased interest and has been validated in several settings and cohorts [24,25,26,27,28,29,30], endorsing its application in routine clinical practice.

In 12,179 critically ill patients hospitalized with congestive HF, Su and colleagues [31] observed that frailty estimated by HFRS was related to in-hospital mortality; similar results were found in pre-frail status and frailty. Short-term mortality (30 days post-discharge) and readmissions were also significantly higher in frail HF patients in a wide cohort analyzed by Kundi and colleagues, who applied the HFRS calculation to 785,127 individuals hospitalized for myocardial infarction, heart failure or pneumonia [32].

Thus, the application of HFRS provides significant evidence, mostly regarding short-term outcome and readmission [30,31,33], but so far, less is known about the role of HFRS as a long-term outcome predictor.

The novelty of our study is the use of HFRS in HF patients to observe long-term readmission risk, following patients up to one year after hospital discharge.

It might be speculated that the early risk of re-hospitalization may be more strongly associated with the acute illness that led to the index hospitalization, which actually has no weight in the calculation of HFRS (calculated based on the patient’s chronic comorbidities, not including the acute conditions developed during hospitalization). In the long term, HFRS might reveal the burden of chronic comorbidities and impairments, even beyond the acute condition that firstly led the patient to hospitalization. Thus, the role of frailty might gain major weight in the long-term perspective and may represent a sort of “baseline risk factor” that influences hospital readmission risk even after the resolution of the acute condition that prompted hospitalization and potentially even beyond the well-known vulnerable phase of HF [2].

Notably, in HF patients, despite adequate adherence to guideline-based HF-specific management strategies, the re-hospitalization rate for HF remains high [6], and our data seem to show that it is influenced by frailty.

These findings have important clinical implications and highlight the need for targeted interventions in frail patients, as well as for tailored follow-up and personalized management plans in individuals with HF. Early identification of frailty among patients hospitalized with heart failure may be broadly applicable, standardized, and reproducible with the use of the HFRS. This approach could help prioritize patients who require specific post-discharge follow-up, potentially influencing not only short-term outcomes but also the long-term risk of hospital readmissions.

Some limitations should be recognized, including the relatively small size of our cohort; moreover, we did not compare the HF subpopulation to patients with different diagnoses. Notably, a proper HF subgroup analysis could not be performed; however, when HF phenotype (preserved vs. reduced EF) was added as a covariate to the Cox model presented in Table 2, it was not significantly associated with hospital readmission risk. All the included patients presented NYHA class 3 or 4; however, further details regarding pre-discharge congestion status would enrich the predictive models regarding hospital readmission risk. Although HFRS displays many advantages in terms of applicability and reproducibility, it should be noted that HFRS based on ICD-10 codes does not stratify the severity of diseases, nor does it include some relevant conditions that might increase the frailty burden regardless. Further studies in larger populations with long follow-up times are warranted to test the validity of HFRS in long-term outcome prediction.

## 5. Conclusions

In conclusion, frailty assessment could allow us to identify HF patients who need to be more closely monitored for a longer time. Compared to other frailty calculation methods, HFRS is broadly feasible and objectively reproducible, potentially in many settings and by different health care professionals, and can be considered a useful and valid screening tool in HF patients.

## Figures and Tables

**Table 1:** Characteristics of the study population.

	Overall (n = 213)		Male (n = 98)		Female (n = 115)		*p*
	Mean	SD	Mean	SD	Mean	SD	
Age (years)	85.70	6.48	84.30	6.47	86.90	6.27	0.004
Weight (kg)	69.40	14.10	71.60	14.80	67.50	13.20	0.035
LOS (days)	12.20	8.12	13.30	9.71	11.30	6.37	0.083
ADL	4.16	2.16	4.82	1.82	3.61	2.28	<0.001
IADL	3.85	3.06	4.38	2.91	3.40	3.13	0.020
MMSE	16.00	10.40	18.50	10.20	13.80	10.10	<0.001
CCI	6.32	1.97	6.68	2.05	6.02	1.85	0.013
Barthel	50.30	30.20	57.20	31.90	44.30	27.30	0.002
HFRS	6.01	2.19	6.01	2.29	6.01	2.11	0.995
SBP (mmHg)	131.00	21.10	129.00	19.50	132.00	22.30	0.343
DBP (mmHg)	73.00	10.20	71.60	9.23	74.20	10.80	0.067
HR (bpm)	82.40	17.80	78.30	15.50	85.90	18.90	0.002
Hb (g/L)	116.00	18.80	114.00	19.30	118.00	18.20	0.085
Creatine (umol/L)	113.00	60.30	129.00	69.00	100.00	48.70	<0.001
eGFR (mL/min/1.73 m^2^)	45.90	22.40	49.80	23.30	42.70	21.10	0.022
NT-proBNP (pg/mL)	9914.00	16,034.00	9614.00	15,235.00	10,170.00	16,747.00	0.802
EF (%)	51.90	13.90	49.70	13.80	54.00	13.80	0.079
Number of medications	7.54	3.33	7.79	3.36	7.33	3.29	0.321
AF (n, %)	85.00	39.91	34.00	34.69	51.00	44.35	0.152
Readmissions (n, %)	117.00	54.93	61.00	62.24	56.00	48.70	0.048
Preserved EF (n, %)	128.00	60.09	51.00	52.04	77.00	66.96	0.008

LOS: length of stay, ADL: activity of daily living, IADL: instrumental activity of daily living, MMSE: mini mental state examination, CCI: Charlson Comorbidity Index, HFRS: hospital frailty risk score, SBP: systolic blood pressure, DBP: diastolic blood pressure, HR: heart rate, Hb: hemoglobin, eGFR: estimated glomerular filtration rate, EF: ejection fraction, AF: atrial fibrillation.

**Table 2:** Cox regression model considering hospital readmissions as dependent variable (R^2^ 0.07).

Variable	HR	95% CI	*p*
Age	0.97	0.94–1.00	0.044
SBP	0.99	0.98–1.00	0.102
NT-proBNP	1.10	0.99–1.21	0.086
HFRS tertiles	1.29	1.03–1.61	0.026

Legend: HFRS: hospital frailty risk score, SBP: systolic blood pressure. NT-proBNP values have been divided by 10,000.

## Data Availability

The data can be made available by the authors upon reasonable request.

## Abbreviations

ADL	activity of daily living
AF	atrial fibrillation
CCI	Charlson Comorbidity Index
DBP	diastolic blood pressure
EF	ejection fraction
eGFR	estimated glomerular filtration rate
Hb	hemoglobin
HF	heart failure
HFRS	hospital frailty risk score
HR	heart rate
IADL	instrumental activity of daily living
LOS	length of stay
MMSE	mini mental state examination
SBP	systolic blood pressure
